# Structured sparse CCA for brain imaging genetics via graph OSCAR

**DOI:** 10.1186/s12918-016-0312-1

**Published:** 2016-08-26

**Authors:** Lei Du, Heng Huang, Jingwen Yan, Sungeun Kim, Shannon Risacher, Mark Inlow, Jason Moore, Andrew Saykin, Li Shen, for the Alzheimer’s Disease Neuroimaging Initiative

**Affiliations:** 1 School of Medicine, Indiana University, Indianapolis, USA; 2 Computer Science & Engineering, University of Texas at Arlington, Arlington, USA; 3 Terre Haute, USA; 4 School of Medicine, University of Pennsylvania, Philadelphia, USA

**Keywords:** Brain imaging genetics, Canonical correlation analysis, Structured sparse model, Machine learning

## Abstract

**Background:**

Recently, structured sparse canonical correlation analysis (SCCA) has received increased attention in brain imaging genetics studies. It can identify bi-multivariate imaging genetic associations as well as select relevant features with desired structure information. These SCCA methods either use the fused lasso regularizer to induce the smoothness between ordered features, or use the signed pairwise difference which is dependent on the estimated sign of sample correlation. Besides, several other structured SCCA models use the group lasso or graph fused lasso to encourage group structure, but they require the structure/group information provided in advance which sometimes is not available.

**Results:**

We propose a new structured SCCA model, which employs the graph OSCAR (GOSCAR) regularizer to encourage those highly correlated features to have similar or equal canonical weights. Our GOSCAR based SCCA has two advantages: 1) It does not require to pre-define the sign of the sample correlation, and thus could reduce the estimation bias. 2) It could pull those highly correlated features together no matter whether they are positively or negatively correlated. We evaluate our method using both synthetic data and real data. Using the 191 ROI measurements of amyloid imaging data, and 58 genetic markers within the *APOE* gene, our method identifies a strong association between *APOE* SNP rs429358 and the amyloid burden measure in the frontal region. In addition, the estimated canonical weights present a clear pattern which is preferable for further investigation.

**Conclusions:**

Our proposed method shows better or comparable performance on the synthetic data in terms of the estimated correlations and canonical loadings. It has successfully identified an important association between an Alzheimer’s disease risk SNP rs429358 and the amyloid burden measure in the frontal region.

## Background

In recent years, the bi-multivariate analyses techniques [1], especially the sparse canonical correlation analysis (SCCA) [2–8], have been widely used in brain imaging genetics studies. These methods are powerful in identifying bi-multivariate associations between genetic biomarkers, e.g., single nucleotide polymorphisms (SNPs), and the imaging factors such as the quantitative traits (QTs).

Witten et al. [3, 9] first employed the penalized matrix decomposition (PMD) technique to handle the SCCA problem which had a closed form solution. This SCCA imposed the *ℓ*_1_-norm into the traditional CCA model to induce sparsity. Since the *ℓ*_1_-norm only randomly chose one of those correlated features, it performed poorly in finding structure information which usually existed in biology data. Witten et al. [3, 9] also implemented the fused lasso based SCCA which penalized two adjacent features orderly. This SCCA could capture some structure information but it demanded the features be ordered. As a result, a lot of structured SCCA approaches arose. Lin et al. [7] imposed the group lasso regularizer to the SCCA model which could make use of the non-overlapping group information. Chen et al. [10] proposed a structure-constrained SCCA (ssCCA) which used a graph-guided fused *ℓ*_2_-norm penalty for one canonical loading according to features’ biology relationships. Du et al. [8] proposed a structure-aware SCCA (S2CCA) to identify group-level bi-multivariate associations, which combined both the covariance matrix information and the prior group information by the group lasso regularizer. These structured SCCA methods, on one hand, can generate a good result when the prior knowledge is well fitted to the hidden structure within the data. On the other hand, they become unapproachable when the prior knowledge is incomplete or not available. Moreover, it is hard to precisely capture the prior knowledge in real world biomedical studies.

To facilitate structural learning via grouping the weights of highly correlated features, the graph theory were widely utilized in sparse regression analysis [11–13]. Recently, we notice that the graph theory has also been employed to address the grouping issue in SCCA. Let each graph vertex and each feature has a one-to-one correspondence relationship, and *ρ*_*ij*_ be the sample correlation between features *i* and *j*. Chen et al. [4, 5] proposed a network-structured SCCA (NS-SCCA) which used the *ℓ*_1_-norm of |*ρ*_*ij*_|(*u*_*i*_−*sign*(*ρ*_*ij*_)*u*_*j*_) to pull those positively correlated features together, and fused those negatively correlated features to the opposite direction. The knowledge-guided SCCA (KG-SCCA) [14] was an extension of both NS-SCCA [4, 5] and S2CCA [8]. It used *ℓ*_2_-norm of $\rho _{ij}^{2}(u_{i}-sign(r_{ij})u_{j})$ for one canonical loading, similar to what Chen proposed, and employed the *ℓ*_2,1_-norm penalty for another canonical loading. Both NS-SCCA and KG-SCCA could be used as a group-pursuit method if the prior knowledge was not available. However, one limitation of both models is that they depend on the sign of pairwise sample correlation to recover the structure pattern. This probably incur undesirable bias since the sign of the correlations could be wrongly estimated due to possible graph misspecification caused by noise [13].

To address the issues above, we propose a novel structured SCCA which neither requires to specify prior knowledge, nor to specify the sign of sample correlations. It will also work well if the prior knowledge is provided. The GOSC-SCCA, named from *G*raph *O*ctagonal *S*election and *C*lustering algorithm for *S*parse *C*anonical *C*orrelation *A*nalysis, is inspired by the outstanding feature grouping ability of octagonal selection and clustering algorithm for regression (OSCAR) [11] regularizer and graph OSCAR (GOSCAR) [13] regularizer in regression task. Our contributions can be summarized as follows 1) GOSC-SCCA could pull those highly correlated features together when no prior knowledge is provided. While those positively correlated features will be encouraged to have similar weights, those negatively correlated ones will also be encouraged to have similar weights but with different signs. 2) Our GOSC-SCCA could reduce the estimation bias given no requirement for specifying the sign of sample correlation. 3) We provide a theoretical quantitative description for the grouping effect of GOSC-SCCA. We use both synthetic data and real imaging genetic data to evaluate GOSC-SCCA. The experimental results show that our method is better than or comparable to those state-of-the-art methods, i.e., L1-SCCA, FL-SCCA [3] and KG-SCCA [14], in identifying stronger imaging genetic correlations and more accurate and cleaner canonical loadings pattern. Note that the PMA software package were used to implement the L1-SCCA (SCCA with lasso penalty) and FL-SCCA (SCCA with fused lasso penalty) methods. Please refer to http://cran.r-project.org/web/packages/PMA/ for more details.

## Methods

We denote a vector as a boldface lowercase letter, and denote a matrix as a boldface uppercase letter. **m**^*i*^ indicates the *i*-th row of matrix **M**=(*m*_*ij*_). Matrices $\mathbf {X} = \{\mathrm {\mathbf {x}}^{1}; \ldots ; \mathrm {\mathbf {x}}^{n}\} \subseteq \mathbb {R}^{p}$ and $\mathbf {Y} = \{\mathrm {\mathbf {y}}^{1}; \ldots ; \mathrm {\mathbf {y}}^{n}\} \subseteq \mathbb {R}^{q}$ denote two separate datasets collected from the same population. Imposing lasso into a traditional CCA model [15], the L1-SCCA model is formulated as follows [3, 9]:

1 $$ \begin{aligned} & \min_{\mathbf{u},\mathbf{v}} -\mathbf{u}^{T} \mathbf{X}^{T} \mathbf{Y} \mathbf{v},\\ & s.t. ||\mathbf{u}||_{2}^{2} = 1, ||\mathbf{v}||_{2}^{2} = 1, ||\mathbf{u}||_{1} \leq c_{1}, ||\mathbf{v}||_{1} \leq c_{2}, \end{aligned}  $$

where ||**u**||_1_≤*c*_1_ and ||**v**||_1_≤*c*_2_ are sparsity penalties controlling the complexity of the SCCA model. The fused lasso [2–4, 9] can also be used instead of lasso. In order to make the problem be convex, the equal sign is usually replaced by less-than-equal sign, i.e. $||\mathbf {u}||_{2}^{2} \leq 1, ||\mathbf {v}||_{2}^{2} \leq 1$ [3].

### The graph OSCAR regularization

The OSCAR regularizer is firstly introduced by Bondell et al. [11], which has been proved to have the ability of grouping features automatically by encouraging those highly correlated features to have similar weights. Formally, the OSCAR penalty is defined as follows,

2 $$ \begin{aligned} ||\mathbf{u}||_{\text{OSCAR}} = \sum\limits_{i<j}\max\{|u_{i}|,|u_{j}|\}, \\ ||\mathbf{v}||_{\text{OSCAR}} = \sum\limits_{i<j}\max\{|v_{i}|,|v_{j}|\}. \end{aligned}  $$

Note that this penalty is applied to each feature pair.

To make OSCAR be more flexible, Yang et al. [13] introduce the GOSCAR,

3 $$ \begin{aligned} ||\mathbf{u}||_{\text{GOSCAR}} = \sum\limits_{(i,j) \in E_{u}}\max\{|u_{i}|,|u_{j}|\}, \\ ||\mathbf{v}||_{\text{GOSCAR}} = \sum\limits_{(i,j) \in E_{v}}\max\{|v_{i}|,|v_{j}|\}. \end{aligned}  $$

where *E*_*u*_ and *E*_*v*_ are the edge sets of the **u**-related and **v**-related graphs, respectively. Obviously, the GOSCAR will reduce to OSCAR when both graphs are complete [13].

Applying $\max \{|u_{i}|,|u_{j}|\} = \frac {1}{2}(|u_{i}-u_{j}|+|u_{i}+u_{j}|)$, the GOSCAR regularizer takes the following form,

4 $${} \begin{aligned} ||\mathbf{u}||_{\text{GOSCAR}} = \frac{1}{2}\sum\limits_{(i,j) \in E_{u}} (|u_{i}-u_{j}|) + \frac{1}{2}\sum\limits_{(i,j) \in E_{u}} (|u_{i}+u_{j}|), \\ ||\mathbf{v}||_{\text{GOSCAR}} = \frac{1}{2}\sum\limits_{(i,j) \in E_{v}} (|v_{i}-v_{j}|) + \frac{1}{2}\sum\limits_{(i,j) \in E_{v}} (|v_{i}+v_{j}|). \end{aligned}  $$

### The GOSC-SCCA model

Since the grouping effect is also an important consideration in SCCA learning, we propose to expand L1-SCCA to GOSC-SCCA by imposing GOSCAR instead of L1 only as follows.

5 $$ \begin{aligned} & \min_{\mathbf{u},\mathbf{v}} -\mathbf{u}^{T} \mathbf{X}^{T} \mathbf{Y} \mathbf{v}\\ & s.t. ~||\mathbf{Xu}||_{2}^{2} \leq 1, ||\mathbf{Yv}||_{2}^{2} \leq 1, ||\mathbf{u}||_{1} \leq c_{1}, ||\mathbf{v}||_{1} \leq c_{2},\\ & ||\mathbf{u}||_{\text{GOSCAR}} \leq c_{3}, ||\mathbf{v}||_{\text{GOSCAR}} \leq c_{4}. \end{aligned}  $$

where (*c*_1_,*c*_2_,*c*_3_,*c*_4_) are parameters and they could control the solution path of the canonical loadings. Since the S2CCA [8] has proved that the covariance matrix information could help improve the prediction ability, we also use $||\mathbf {Xu}||_{2}^{2} \leq 1$ and $||\mathbf {Yv}||_{2}^{2} \leq 1$ other than $||\mathbf {u}||_{2}^{2} \leq 1, ||\mathbf {v}||_{2}^{2} \leq 1$.

As a structured sparse model, GOSC-SCCA will encourage $u_{i} \doteq u_{j}$ if the *i*-th feature and the *j*-th feature are highly correlated. We will give a quantitative description for this later.

### The proposed algorithm

We can write the objective function into unconstrained formulation via the Lagrange multiplier method, i.e.

6 $${} \begin{aligned} \mathbf{\mathcal{L}(u,v)} &= -\mathbf{u}^{T} \mathbf{X}^{T} \mathbf{Y} \mathbf{v} + \lambda_{1}||\mathbf{u}||_{\text{GOSCAR}}+\lambda_{2}||\mathbf{v}||_{\text{GOSCAR}} \\ &\quad +\frac{\beta_{1}}{2}||\mathbf{u}||_{1}+\frac{\beta_{2}}{2}||\mathbf{v}||_{1}+ +\frac{\gamma_{1}}{2}||\mathbf{Xu}||_{2}^{2}+\frac{\gamma_{2}}{2}||\mathbf{Yv}||_{2}^{2} \end{aligned}  $$

where (*λ*_1_,*λ*_2_,*β*_1_,*β*_2_) are tuning parameters, and they have a one-to-one correspondence to parameters (*c*_1_,*c*_2_,*c*_3_,*c*_4_) in GOSC-SCCA model [4].

Taking the derivative regarding **u** and **v** respectively, and letting them be zero, we obtain,

7 $$\begin{array}{@{}rcl@{}} -\mathbf{X}^{T}\mathbf{Yv} +\lambda_{1} \mathbf{L}_{1}\mathbf{u} +\lambda_{1} \hat{\mathbf{L}}_{1}\mathbf{u}+ \beta_{1}\mathbf{\Lambda_{1}} +\gamma_{1}\mathbf{X}^{T}\mathbf{X}\mathbf{u}=0, \end{array} $$

8 $$\begin{array}{@{}rcl@{}}  -\mathbf{Y}^{T}\mathbf{Xu}+\lambda_{2} \mathbf{L}_{2}\mathbf{v}+\lambda_{2} \hat{\mathbf{L}}_{2}\mathbf{v}+\beta_{2}\mathbf{\Lambda_{2}}+\gamma_{2}\mathbf{Y}^{T}\mathbf{Y}\mathbf{v}=0. \end{array} $$

where *Λ*_1_ is a diagonal matrix with the *k*_1_-th element as $\frac {1}{2||u_{k_{1}}||_{1}} (k_{1} \in [1,p])$, and *Λ*_2_ with the *k*_2_-th element as $\frac {1}{2||v_{k_{2}}||_{1}} (k_{2} \in [1,q])$; **L**_1_ is the Laplacian matrix which can be obtained from **L**_1_=**D**_1_−**W**_1_; $\hat {\mathbf {L}}_{1}$ is a matrix which is from $\hat {\mathbf {L}}_{1} = \hat {\mathbf {D}}_{1} + \hat {\mathbf {W}}_{1}$. **L**_2_ and $\hat {\mathbf {L}}_{2}$ have the same entries as **L**_1_ and $\hat {\mathbf {L}}_{1}$ separately based on **v**.

In the initialization, both **W**_1_ and $\hat {\mathbf {W}}_{1}$ have the same entry with each element as $\frac {1}{2}$ except the diagonal elements. But **W**_1_ and $\hat {\mathbf {W}}_{1}$ become different after each iteration, i.e.,

9 $$ w_{ij} = \frac{1}{2|u_{i}-u_{j}|}, ~~~{\hat{w}}_{ij} = \frac{1}{2|u_{i}+u_{j}|}.  $$

If ||*u*_*i*_−*u*_*j*_||_1_=0, the corresponding element in matrix **W**_1_ will not exist. So we regularize it as $\frac {1}{2\sqrt {||u_{i}-u_{j}||_{1}^{2}+\zeta }}$ (*ζ* is a very small positive number) when ||*u*_*i*_−*u*_*j*_||_1_=0. We also approximate ||*u*_*i*_||_1_=0 with $\sqrt {||u_{i}||_{1}^{2}+\zeta }$ for *Λ*_1_. Then the objective function regarding **u** is $\mathbf {\mathcal {L^{*}}(u)} = \sum _{i=1}^{p} (-u^{i} \mathbf {x}_{i}^{T} \mathbf {Y} \mathbf {v} + \lambda _{1}\sum || \sqrt {||u_{i}||_{1}^{2}+\zeta }||_{\text {GOSCAR}}+\frac {\beta 1}{2}\sqrt {||u_{i}||_{1}^{2}+\zeta } +\frac {\gamma _{1}}{2}||\mathbf {x}_{i}u_{i}||_{2}^{2})$. It is easy to prove that $\mathcal {L^{*}}(\mathbf {u})$ will reduce to problem (6) regarding **u** when *ζ*→0. The cases of ||*v*_*i*_||_1_=0 and ||*v*_*i*_−*v*_*j*_||_1_=0 can be addressed using a similar regularization method.

**D**_1_ is a diagonal matrix and its *i*-th diagonal element is obtained by summing the *i*-th row of **W**_1_, i.e. $d_{i} = \sum _{j} w_{ij}$. The diagonal matrix $\hat {\mathbf {D}}_{1}$ is also obtained from ${\hat {d}}_{i} = \sum _{j} {\hat {w}}_{ij}$. Likewise, we can calculate **W**_2_, $\hat {\mathbf {W}}_{2}$, **D**_2_ and $\hat {\mathbf {D}}_{2}$ by the same method in terms of **v**.

Then according to Eqs. (7-8), we can obtain the solution to our problem with respect to **u** and **v** separately.

10 $$\begin{array}{@{}rcl@{}} \mathbf{u}=(\lambda_{1} (\mathbf{L}_{1}+\hat{\mathbf{L}}_{1}) +\beta_{1}\mathbf{\Lambda_{1}}+\gamma_{1}\mathbf{X^{T}X})^{-1}\mathbf{X^{T}Yv}, \end{array} $$

11 $$\begin{array}{@{}rcl@{}}  \mathbf{v}=(\lambda_{2} (\mathbf{L}_{2}+\hat{\mathbf{L}}_{2}) +\beta_{2}\mathbf{\Lambda_{2}}+\gamma_{2}\mathbf{Y^{T}Y})^{-1}\mathbf{Y^{T}Xu}. \end{array} $$

We observe that **L**_1_, $\hat {\mathbf {L}}_{1}$ and *Λ*_1_ depend on **u** which is an unknown variable, and **v** is also unknown which is used to calculate **L**_2_, $\hat {\mathbf {L}}_{2}$ and *Λ*_2_. Thus we propose an effective iterative algorithm to solve this problem. We first fix **v** to solve **u**; and then fix **u** to solve **v**.

Algorithm 1 exhibits the pseudo code of the proposed GOSC-SCCA algorithm. For the key calculation steps, i.e., Step 5 and Step 10, we solve a system of linear equations with quadratic complexity other than computing the matrix inverse with cubic complexity. Thus the whole algorithm can work with desired efficiency. In addition, the algorithm is guaranteed to converge and we will prove this in the next subsection.

### Convergence analysis

We first introduce the following lemma.

#### **Lemma 1**

For any two nonzero real numbers $\tilde {u}$ and *u*, we have

12 $$  ||\tilde{u}||_{1}-\frac{||\tilde{u}||_{1}^{2}}{2||u||_{1}} \leq ||u||_{1}-\frac{||u||_{1}^{2}}{2||u||_{1}}.  $$

#### *Proof*

Given the lemma in [16], we have $||\tilde {\mathbf {u}}||_{2}-\frac {||\tilde {\mathbf {u}}||_{2}^{2}}{2||\mathbf {u}||_{2}} \leq ||\mathbf {u}||_{2}-\frac {||\mathbf {u}||_{2}^{2}}{2||\mathbf {u}||_{2}}$ for any two nonzero vectors. We also have $||\tilde {u}||_{1}=||\tilde {u}||_{2}$ and ||*u*||_1_=||*u*||_2_ for any two nonzero real numbers, which completes the proof. □

Based on Lemma 1, we have

13 $$\begin{array}{@{}rcl@{}} \!\!\!\! ||\tilde{u}' - u'||_{1}-\frac{||\tilde{u}' - u'||_{1}^{2}}{2||\tilde{u} - u||_{1}} \leq ||\tilde{u} - u||_{1}-\!\frac{||\tilde{u} - u||_{1}^{2}}{2||\tilde{u} - u||_{1}}, \end{array} $$

14 $$\begin{array}{@{}rcl@{}}  \!\!\!\! ||\tilde{u}' + u'||_{1}-\frac{||\tilde{u}' + u'||_{1}^{2}}{2||\tilde{u} + u||_{1}} \leq ||\tilde{u} + u||_{1}-\!\frac{||\tilde{u} + u||_{1}^{2}}{2||\tilde{u} + u||_{1}}, \end{array} $$

when $|\tilde {u}' - u'|$, $|\tilde {u} - u|$, $|\tilde {u}' + u'|$ and $|\tilde {u} + u|$ are nonzero.

We now have the following theorem regarding GOSC-SCCA algorithm.

#### **Theorem 1**

The objective function value of GOSC-SCCA will monotonically decrease in each iteration till the algorithm converges.

#### *Proof*

The proof consists of two parts.

(1) Part 1: From Step 3 to Step 7 in Algorithm 1, **u** is the only unknown variable to be solved. The objective function (6) can be equivalently transferred to

$${} \mathbf{\mathcal{L}(\!u,v\!)} = -\mathbf{u}^{T} \mathbf{X}^{T} \mathbf{Y} \mathbf{v} + \lambda_{1}||\mathbf{u}||_{\text{GOSCAR}}+\frac{\beta_{1}}{2}||\mathbf{u}||_{1}+\frac{\gamma_{1}}{2}||\mathbf{Xu}||_{2}^{2} $$

According to Step 5 we have

$$\begin{aligned} & -\tilde{\mathbf{u}}^{\mathbf{T}}\mathbf{X^{T}Yv}+\lambda_{1}\tilde{\mathbf{u}}^{\mathbf{T}}\tilde{\mathbf{L}}_{\mathbf{1}}\tilde{\mathbf{u}} +\lambda_{1}\tilde{\mathbf{u}}^{\mathbf{T}}{\tilde{\hat{\mathbf{L_1}}}}\tilde{\mathbf{u}}\\ &+\beta_{1}\tilde{\mathbf{u}}^{\mathbf{T}} \mathbf{\Lambda_{1}} \tilde{\mathbf{u}}+\gamma_{1}\tilde{\mathbf{u}}^{\mathbf{T}}\mathbf{X^{T}X}\tilde{\mathbf{u}}\\ & \leq -\mathbf{u^{T}X^{T}Yv}+\lambda_{1}\mathbf{u^{T}}\mathbf{L_{1}}\mathbf{u} +\lambda_{1}\mathbf{u^{T}}\hat{\mathbf{L_{1}}}\mathbf{u}\\ &+\beta_{1}\mathbf{u^{T}\Lambda_{1} u}+\gamma_{1}\mathbf{u^{T}X^{T}Xu} \end{aligned} $$

where $\tilde {\mathbf {u}}$ is the updated **u**.

It is known that $\mathbf {u^{T}}\mathbf {L}\mathbf {u} = \sum w_{ij} ||u_{i}-u_{j}||_{1}^{2}$ if **L** is the laplacian matrix [17]. Similarly, $\mathbf {u^{T}}\hat {\mathbf {L}}\mathbf {u} = \sum w_{ij} ||u_{i}+u_{j}||_{1}^{2}$. Then according to Eq. (9), we obtain

15 $${} \begin{aligned} & -\tilde{\mathbf{u}}^{\mathbf{T}}\mathbf{X^{T}Yv}+2\lambda_{1}\sum w_{ij}\frac{||\tilde{u}_{i}-\tilde{u}_{j}||_{1}^{2}}{2||u_{i}-u_{j}||_{1}}\\ & +2\lambda_{1}\sum {\hat{w}}_{ij}\frac{||\tilde{u}_{i}+\tilde{u}_{j}||_{1}^{2}}{2||u_{i}+u_{j}||_{1}}+\beta_{1}\sum \frac{||\tilde{u}_{i}||_{1}^{2}}{2||u_{i}||_{1}}+\gamma_{1}\tilde{\mathbf{u}}^{\mathbf{T}}\mathbf{X^{T}X}\tilde{\mathbf{u}}\\ & \leq -\mathbf{u^{T}X^{T}Yv}+2\lambda_{1}\sum w_{ij} \frac{||u_{i}-u_{j}||_{1}^{2}}{2||u_{i}-u_{j}||_{1}}+\\ & 2\lambda_{1}\sum {\hat{w}}_{ij} \frac{||u_{i}+u_{j}||_{1}^{2}}{2||u_{i}+u_{j}||_{1}} +\beta_{1}\sum \frac{||u_{i}||_{1}^{2}}{2||u_{i}||_{1}}+\gamma_{1}\mathbf{u^{T}X^{T}Xu} \end{aligned}  $$

We first multiply 2*λ*_1_ on both sides of Eq. (13) for each feature pair separately, and do the same to both sides of Eq. (14). After that, we multiply *β*_1_ on both sides of Eq. (12). Finally, by summing all these inequations together to both sides of Eq. (15) accordingly, we arrive at

$${} \begin{aligned} & -\tilde{\mathbf{u}}^{\mathbf{T}}\mathbf{X^{T}Yv}+2\lambda_{1} \sum w_{ij}|{\tilde{u}}_{i}- {\tilde{u}}_{j}|+2\lambda_{1} \sum {\hat{w}}_{ij}|{\tilde{u}}_{i} + {\tilde{u}}_{j}|\\ & +\beta_{1}||\tilde{\mathbf{u}}||_{1}+\gamma_{1}||{\mathbf{X}}{\tilde{\mathbf{u}}}||_{2}^{2} \\ & \leq -\mathbf{u^{T}X^{T}Yv}+2\lambda_{1}\sum w_{ij}|u_{i}-u_{j}|+2\lambda_{1}\sum {\hat{w}}_{ij}|u_{i}+u_{j}| \\ & +\beta_{1}||\mathbf u||_{1}+\gamma_{1}||\mathbf{Xu}||_{2}^{2}. \end{aligned} $$

Let $\lambda _{1}^{*} = 2\lambda _{1}$, $\gamma _{1}^{*} = 2\gamma _{1},\beta _{1}^{*} = 2\beta _{1}$, we have

16 $${} \begin{aligned} -\tilde{\mathbf{u}}^{\mathbf{T}}\mathbf{X^{T}Yv}+\frac{\lambda_{1}^{*}}{2}||\tilde{\mathbf{u}}||_{\text{GOSCAR}}+\frac{\beta_{1}^{*}}{2}||\tilde{\mathbf{u}}||_{1}+\frac{\gamma_{1}^{*}}{2}||\mathbf{X}\tilde{\mathbf{u}}||_{2}^{2} \\ \leq -\mathbf{u^{T}X^{T}Yv}+\frac{\lambda_{1}^{*}}{2}||\mathbf{u}||_{\text{GOSCAR}}+\frac{\beta_{1}^{*}}{2}||\mathbf{u}||_{1}+\frac{\gamma_{1}^{*}}{2}\mathbf{||Xu||}_{2}^{2}. \end{aligned}  $$

Therefore, GOSC-SCCA will decrease the objective function in each iteration, i.e., $\mathbf {\mathcal {L}(}\tilde {\mathbf {u}}\mathbf {,v)} \leq \mathbf {\mathcal {L}(u,v)}$.

(2) Part 2: From Step 8 to Step 12, the only unknown variable is **v**. Similarly, we can arrive at

17 $${} \begin{aligned} -\tilde{\mathbf{u}}^{\mathbf{T}}\mathbf{X^{T}Y} \tilde{\mathbf{v}}+\frac{\lambda_{2}^{*}}{2}||\tilde{\mathbf{v}}||_{\text{GOSCAR}}+\frac{\beta_{2}^{*}}{2}||\tilde{\mathbf{v}}||_{1}+\frac{\gamma_{2}^{*}}{2}||\mathbf{Y}\tilde{\mathbf{v}}||_{2}^{2} \\ \leq -\tilde{\mathbf{u}}^{\mathbf{T}}\mathbf{X^{T}Yv}+\frac{\lambda_{2}^{*}}{2}||\mathbf{v}||_{\text{GOSCAR}}+\frac{\beta_{2}^{*}}{2}||\mathbf{v}||_{1}+\frac{\gamma_{2}^{*}}{2}||\mathbf{Yv}||_{2}^{2}. \end{aligned}  $$

Thus GOSC-SCCA also decreases the objective function in each iteration during the second phase, i.e., $\mathbf {\mathcal {L}}(\tilde {\mathbf {u}},\tilde {\mathbf {v}}) \leq \mathbf {\mathcal {L}}(\tilde {\mathbf {u}},\mathbf {v})$.

Based on the analysis above, we easily have $\mathbf {\mathcal {L}}(\tilde {\mathbf {u}},\tilde {\mathbf {v}}) \leq \mathbf {\mathcal {L}(u,v)}$ according to the transitive property of inequalities. Therefore, the objective value monotonically decreases in each iteration. Note that the CCA objective $\mathbf {\frac {u^{T}X^{T}Yv}{\sqrt {u^{T}X^{T}Xu}\sqrt {v^{T}Y^{T}Yv}}}$ ranges from [-1,1], and both *u*^*T*^*X*^*T*^**Xu** and *v*^*T*^*Y*^*T*^**Yv** are constrained to be 1. Thus the −*u*^*T*^*X*^*T*^**Yv** is lower bounded by -1, and so Eq. (6) is lower bounded by –1. In addition, Eqs. (16–17) imply that the KKT condition is satisfied. Therefore, the GOSC-SCCA algorithm will converge to a local optimum. □

Based on the convergence analysis, to facilitate the GOSC-SCCA algorithm, we set the stopping criterion of Algorithm 1 as max{|*δ*|∣*δ*∈(**u**_*t*+1_−**u**_*t*_)}≤*τ* and max{|*δ*|∣*δ*∈(**v**_*t*+1_−**v**_*t*_)}≤*τ*, where *τ* is a predefined estimation error. Here we set *τ*=10^−5^ empirically from the experiments.

### The grouping effect of GOSC-SCCA

For the structured sparse learning in high-dimensional situation, the *automatic feature grouping* property is of great importance [18]. In regression analysis, Zou and Hastie [18] have suggested that a regressor behaviors grouping effect when it can set those regression coefficients of the same group to similar weights. This is also the case for structured SCCA methods. So, it is important and meaningful to investigate the theoretical boundary of the grouping effect.

We have the following theorem in terms of GOSC-SCCA.

#### **Theorem 2**

Let **X** and **Y** be two data sets, and (*λ*,*β*,*γ*)be the pre-tuned parameters. Let $\tilde {\mathbf {u}}$ be the solution to our SCCA problem of Eqs. (10–11). Suppose the *i*-th feature and *j*-th feature only link to each other on the graph, $\tilde {u}_{i}$ and $\tilde {u}_{j}$ are their optimal solutions, thus $\text {sgn}(\tilde {u}_{i}) = \text {sgn}(\tilde {u}_{j})$holds. The solutions to $\tilde {u}_{i}$ and $\tilde {u}_{j}$ satisfy

18 $$ |\tilde{u}_{i}-\tilde{u}_{j}| \leq \frac{2\lambda_{1} w_{ij}}{\gamma_{1}}+ \frac{1}{\gamma_{1}}\sqrt{2(1-\rho_{ij})}  $$

where *ρ*_*ij*_ is the sample correlation between features *i* and *j*, and *w*_*i*,*j*_ is the corresponding element in **u**-related matrix **W**_1_.

#### *Proof*

Let $\tilde {\mathbf {u}}$ be the solution to our problem Eq. (6), we have the following equations after taking the partial derivative with respect to $\tilde u_{i}$ and $\tilde u_{j}$, respectively.

$$\begin{aligned} (\lambda_{1} \mathbf{L}_{1}^{i}+\lambda_{1} {\hat{\mathbf{L}}}_{1}^{i} +\beta_{1}{\Lambda_{1}}_{ii}+\gamma_{1}\mathbf{x}_{i}^{T}\mathbf{x}_{i})\tilde{u}_{i}=\mathbf{x}_{i}^{T}\mathbf{Yv},\\ (\lambda_{1} \mathbf{L}_{1}^{j}+\lambda_{1} {\hat{\mathbf{L}}}_{1}^{j} +\beta_{1}{\Lambda_{1}}_{jj}+\gamma_{1}\mathbf{x}_{j}^{T}\mathbf{x}_{j})\tilde{u}_{j}=\mathbf{x}_{j}^{T}\mathbf{Yv}. \end{aligned} $$

We know that features *i* and *u*_*j*_ are only linked to each other, thus *D*_*ii*_=*D*_*jj*_=*A*_*ij*_=*w*_*ij*_ for those intermediate matrices. Besides, we also know that $\text {sgn}(\tilde u_{i})=\frac {\tilde u_{i}}{|\tilde u_{i}|}$, $\text {sgn}(\tilde u_{i})=\text {sgn}(\tilde u_{j})$, $\mathbf {x}_{i}^{T}\mathbf {x}_{i} = \rho _{ii} = 1$ and $\mathbf {x}_{j}^{T}\mathbf {x}_{j} = \rho _{jj} = 1$. Then according to the definition of **L**_1_, ${\hat {\mathbf {L}}}_{1}$ and **Λ**_1_, we can arrive at

19 $${} \begin{aligned} \lambda_{1} w_{ij}\text{sgn}(\tilde{u}_{i}-\tilde{u}_{j}) + \lambda_{1} {\hat{w}}_{ij}\text{sgn}(\tilde{u}_{i}+\tilde{u}_{j}) +\beta_{1}\text{sgn}(\tilde{u}_{i})+\gamma_{1}\tilde{u}_{i}\\ =\mathbf{x}_{i}^{T}\mathbf{Yv},\\ \lambda_{1} w_{ij}\text{sgn}(\tilde{u}_{j}-\tilde{u}_{i}) + \lambda_{1} {\hat{w}}_{ij}\text{sgn}(\tilde{u}_{i}+\tilde{u}_{j}) +\beta_{1}\text{sgn}(\tilde{u}_{j})+\gamma_{1}\tilde{u}_{j}\\ =\mathbf{x}_{j}^{T}\mathbf{Yv}. \end{aligned}  $$

Subtracting these two equations, we obtain

20 $$  \begin{aligned} \gamma_{1}(\tilde{u}_{i}-\tilde{u}_{j}) = 2\lambda_{1}w_{ij}\text{sgn}(\tilde{u}_{j}-\tilde{u}_{i}) + (\mathbf{x}_{i}-\mathbf{x}_{j})^{T}\mathbf{Yv} \end{aligned}  $$

Then we take *ℓ*_2_-norm on both sides of Eq. (20), apply the triangle inequality, and use the equality $||(\mathbf {x}_{i}-\mathbf {x}_{j})||_{2}^{2} = 2(1-\rho _{ij})$,

21 $$ \begin{aligned} \gamma_{1}|\tilde{u}_{i}-\tilde{u}_{j}| \leq 2\lambda_{1}w_{ij} + \sqrt{2(1-\rho_{ij})}\sqrt{||\mathbf{Yv}||_{2}^{2}} \end{aligned}  $$

We have known that our problem implies $||\mathbf {Yv}||_{2}^{2} \leq 1$, thus we arrive at

22 $$ \begin{aligned} |\tilde{u}_{i}-\tilde{u}_{j}| \leq \frac{2\lambda_{1} w_{ij}}{\gamma_{1}}+ \frac{1}{\gamma_{1}}\sqrt{2(1-\rho_{ij})} \end{aligned}  $$

□

Now the upper bound for the canonical loadings **v** can also be obtained, i.e.

23 $$ \begin{aligned} |\tilde{v}_{i}-\tilde{v}_{j}| \leq \frac{2\lambda_{2} w'_{ij}}{\gamma_{2}}+ \frac{1}{\gamma_{2}}\sqrt{2(1-\rho'_{ij})} \end{aligned}  $$

where *ρij*′ is the sample correlation between the *i*-th and *j*-th feature in **v**, and $w^{\prime }_{ij}$ is the corresponding element in **v**-related matrix **W**_2_.

Theorem 2 provides a theoretical upper bound for the difference between the estimated coefficients of the *i*-th feature and *j*-th feature. It seems that this is not a tight enough bound. However our bound is slack since it does not bound much more the pairwise difference of features *i* and *j* if *ρ*_*ij*_≪1. This is desirable for two irrelevant features [19]. Suppose two features with very small correlation, i.e. *ρ*_*ij*_≪0, their coefficients do not need to be the same or similar. So we do not care about their coefficients’ pairwise difference, and will not set their pairwise difference a tight bound. This quantitative description for the grouping effect makes the GOSCAR penalty an ideal choice for structured SCCA.

## Results

We compare GOSC-SCCA with several state-of-the-art SCCA and structured SCCA methods, including L1-SCCA [3], FL-SCCA [3], KG-SCCA [14]. We do not compare GOSC-SCCA with S2CCA [8], ssCCA [7] and CCA-SG (CCA Sparse Group) [10] since they require prior knowledge available in advance. We do not choose NS-SCCA [5] as benchmark either, due to the following two reasons. (1) NS-SCCA generates many intermediate variables during its iterative procedure. As the authors stated, NS-SCCA’s per-iteration complexity is linear in (*p*+|*E*|), and thus the complexity becomes *O*(*p*^2^) when it is in the group pursuit mode. (2) Its penalty term is similar to that of KG-SCCA which has been selected for comparison.

There are six parameters to be decided before using the GOSC-SCCA, thus it will take too much time by blindly tuning. We tune the parameters following two principles. On one hand, Chen and Liu [5] found out that the result is not very sensitive to *γ*_1_ and *γ*_2_. So we choose them from a small scope [0.1, 1, 10]. On the other hand, if the parameters are too small, the SCCA will reduce to CCA due to the subtle influence of the penalties. And, too large parameters will over-penalize the results. Therefore, we tune the rest of the parameters within the range of {10^−3^,10^−2^,10^−1^,10^0^,10^1^,10^2^,10^3^}. In this study, we conduct all the experiments using the **nested** 5-fold cross-validation strategy, and the parameters are only tuned from the training set. In order to save time, we only tune these parameters on the first run of the cross-validation. That is, the parameters are tuned when the first four folds are used as the training set. Then we directly use the tuned parameters for all the remaining experiments. All these methods use the same partition for cross-validation in the experiment.

### Evaluation on synthetic data

We generate four synthetic datasets to investigate the performance of GOSC-SCCA and those benchmarks. Following [4, 5], these datasets are generated by four steps: 1) We predefine the structures and use them to create **u** and **v** respectively. 2) We create a latent vector **z** from *N*(**0**,**I**_*n*×*n*_). 3) We create **X** with each $\mathbf {x}_{i} \sim N(z_{i}\mathbf {u},\sum _{x})$ where $(\sum _{x})_{jk}=\exp ^{-|u_{j}-u_{k}|}$ and **Y** with each $\mathbf {y}_{i} \sim N(z_{i}\mathbf {v},\sum _{y})$ where $(\sum _{y})_{jk}=\exp ^{-|v_{j}-v_{k}|}$. 4) For the first group of nonzero features in **u**, we change half of their signs, and also change the signs of the corresponding data. Since the synthetic datasets are order-independent, this setup is equivalent to randomly change a portion of features’ signs in **u**. Now that we change the sign of both coefficients and the data simultaneously, we still have *X*^′^*u*^′^=*Xu* where *X*^′^ and *u*^′^ indicate the data and coefficients after the sign swap. We do the same on the **Y** side to make our simulation more challenging [13]. In addition, we set all four datasets with *n*=80, *p*=100 and *q*=120. They also have different correlation coefficients and different group structures. Therefore, the simulation is designed to cover a set of diverse cases for a fair comparison.

The estimated correlation coefficients of each method on four datasets are contained in Table 1. The best values and those are not significantly worsen than the best values are shown in bold. On the training results, we observe that GOSC-SCCA either estimates the largest correlation coefficients (Dataset 1 and Dataset 4), or is not significantly worse than the best method (Dataset 2 and Dataset 3). GOSC-SCCA also has the best average correlation coefficients. On the testing results, GOSC-SCCA also outperforms those benchmarks in terms of the average correlation coefficients, though KG-SCCA does not perform significantly worse than our method. For the overall average obtained across four datasets, GOSC-SCCA obtains the better correlation coefficients than the competing methods on both training set and testing set.

**Table 1:** 5-fold cross-validation results on synthetic data

Training results																									
Methods			Dataset 1			MEAN			Dataset 2			MEAN			Dataset 3			MEAN			Dataset 4			MEAN	AVG.
L1-SCCA	0.52	0.56	0.52	0.53	0.51	0.53	0.25	0.29	0.16	0.20	0.23	**0.23**	0.56	0.24	0.57	0.53	0.52	**0.48**	0.46	0.50	0.53	0.48	0.35	0.46	0.43
FL-SCCA	0.52	0.60	0.52	0.53	0.50	0.53	NaN	NaN	0.17	NaN	0.23	0.08	0.63	0.43	0.56	0.55	0.55	**0.54**	0.51	0.56	NaN	0.53	0.40	0.40	0.39
KG-SCCA	0.52	0.55	0.52	0.53	0.53	0.53	0.25	0.29	0.15	0.20	0.22	**0.22**	0.56	0.24	0.43	0.52	0.52	0.45	0.51	0.56	0.48	0.52	0.40	**0.49**	0.42
GOSC-SCCA	0.57	0.62	0.57	0.59	0.63	**0.60**	0.26	0.30	0.15	0.21	0.17	**0.22**	0.64	0.31	0.42	0.61	0.59	**0.51**	0.51	0.56	0.55	0.54	0.41	**0.52**	**0.46**
Testing results																									
L1-SCCA	0.57	0.43	0.58	0.49	0.59	**0.53**	0.00	0.21	0.32	0.17	0.08	**0.16**	0.36	0.20	0.37	0.49	0.46	**0.38**	0.45	0.29	0.20	0.40	0.67	0.40	0.37
FL-SCCA	0.56	0.38	0.57	0.49	0.59	**0.52**	NaN	NaN	0.48	NaN	0.08	0.11	0.30	0.80	0.36	0.51	0.41	**0.47**	0.55	0.30	NaN	0.46	0.72	0.40	0.38
KG-SCCA	0.56	0.43	0.57	0.49	0.58	**0.53**	0.00	0.21	0.31	0.18	0.07	**0.15**	0.37	0.20	0.45	0.50	0.45	**0.39**	0.52	0.29	0.34	0.46	0.71	**0.46**	0.38
GOSC-SCCA	0.73	0.39	0.68	0.56	0.45	**0.56**	0.02	0.09	0.57	0.20	0.38	**0.25**	0.23	0.18	0.43	0.44	0.43	**0.34**	0.53	0.31	0.31	0.36	0.72	**0.45**	**0.40**

The estimated correlation coefficients and their MEAN are shown. ’NaN’ means a method fails to estimate a pair of canonical loadings. ’0.00’ means a very small correlation coefficients. ’AVG.’ denotes the MEAN across all four datasets. The best values and those that are NOT significantly worse than the best ones (*t*-test with *p*-value smaller than 0.05) are shown in bold

**Fig. 1:**
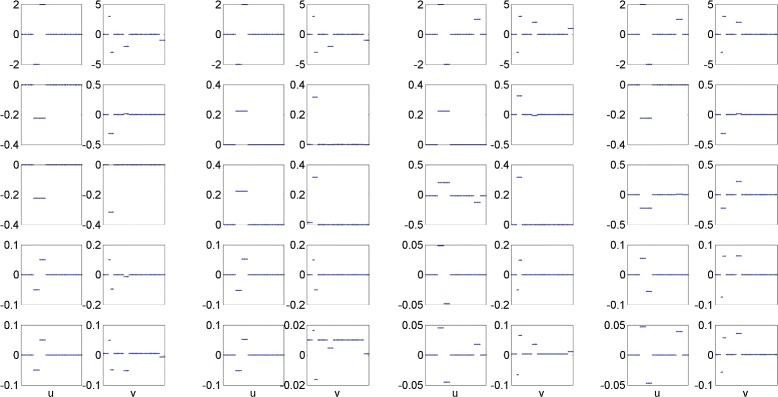
Canonical loadings estimated on four synthetic datasets. The first column is for Dataset 1, and the second column is for Dataset 2, and so forth. For each dataset, the weights of **u** are shown on the left panel, and those of **v** are on the right. The first row is the ground truth, and each remaining row corresponds to a specific method: (1) Ground Truth. (2) L1-SCCA. (3) FL-SCCA. (4) KG-SCCA. (5) GOSC-SCCA

Figure 1 shows the estimated canonical loadings of all four SCCA methods in a typical run. As we can see, L1-SCCA cannot accurately recover the true signals. For those coefficients with sign swapped, it fails to recognize them. The FL-SCCA slightly improves L1-SCCA’s performance but cannot identify those coefficients with sign changed either. Our GOSC-SCCA successfully groups those nonzero features together, and accurately recognizes the coefficients whose signs are changed. No matter what structures are within the dataset, GOSC-SCCA is able to estimate true signals which are very close to the ground truth. Although KG-SCCA also recognizes the coefficients with sign swapped, it is unable to recover every group of nonzero coefficients. For example, KG-SCCA misses two groups of nonzero features in terms of **v** for the second dataset. The results on synthetic datasets reveal that GOSC-SCCA can not only estimate stronger correlation coefficients than the competing methods, but also identifies more accurate and cleaner canonical loadings.

### Evaluation on real neuroimaging genetics data

Data used in the preparation of this article were obtained from the Alzheimer’s Disease Neuroimaging Initiative (ADNI) database (adni.loni.usc.edu). The ADNI was launched in 2003 as a public-private partnership, led by Principal Investigator Michael W. Weiner, MD. The primary goal of ADNI has been to test whether serial magnetic resonance imaging (MRI), positron emission tomography (PET), other biological markers, and clinical and neuropsychological assessment can be combined to measure the progression of mild cognitive impairment (MCI) and early Alzheimer’s disease (AD). For up-to-date information, see www.adni-info.org.

Table 2 contains the characteristics of the ADNI dataset used in this work. Participants including 568 non-Hispanic Caucasian subjects, including 196 healthy control (HC), 343 MCI and 28 AD participants. However, many participants’s data are incomplete due to various factors such as data loss. After cleaning those participants with incomplete information, we get 282 participants in our experiments. The genotype data were downloaded from LONI (adni.loni.usc.edu), and the preprocessed [11C] Florbetapir PET scans (i.e., amyloid imaging data) were also obtained from LONI. Before conducting the experiment, the amyloid imaging data had been preprocessed and the specific pipeline could be found in [14]. These imaging measures were adjusted by removing the effects of the baseline age, gender, education, and handedness via the regression weights derived from HC participants. We finally obtained 191 region-of-interest (ROI) level amyloid measurements which were extracted from the MarsBaR AAL atlas. We included four genetic markers, i.e., rs429358, rs439401, rs445925 and rs584007, from the known AD risk gene *APOE*. We intend to investigate if our GOSC-SCCA could identify this widely known associations between amyloid deposition and *APOE* SNPs.

**Table 2:** Real data characteristics

	HC	MCI	AD
Num	196	343	28
Gender(M/F)	102/94	203/140	18/10
Handedness(R/L)	178/18	309/34	23/5
Age (mean ±std.)	74.77 ±5.39	71.92 ±7.47	75.23 ±10.66
Education (mean ±std.)	15.61 ±2.74	15.99 ±2.75	15.61 ±2.74

Shown in Table 3 are the 5-fold cross-validation results of various SCCA methods. We observe that GOSC-SCCA and KG-SCCA obtain similar correlation coefficients on every run, including the training performance and testing performance. Besides, they both are significantly better than L1-SCCA and FL-SCCA, which is consistent with the analysis in [14]. This result shows that GOSC-SCCA can improve the ability of identifying interesting imaging genetic associations compared with L1-SCCA and FL-SCCA.

**Table 3:** 5-fold cross-validation results on real data

Methods	Training results					MEAN	Testing results					MEAN
L1-SCCA	0.50	0.50	0.53	0.53	0.54	0.52	0.56	0.61	0.45	0.47	0.38	0.49
FL-SCCA	0.44	0.43	0.46	0.45	0.46	0.45	0.49	0.56	0.39	0.43	0.37	0.45
KG-SCCA	0.53	0.52	0.55	0.54	0.56	**0.54**	0.56	0.61	0.47	0.52	0.45	**0.52**
GOSC-SCCA	0.53	0.52	0.55	0.55	0.56	**0.54**	0.56	0.62	0.47	0.51	0.45	**0.52**

The estimated correlation coefficients and their MEAN are shown. The best correlation coefficients and those that are NOT significantly worse than the best ones (*t*-test with *p*-value smaller than 0.05) are shown in bold

**Fig. 2:**
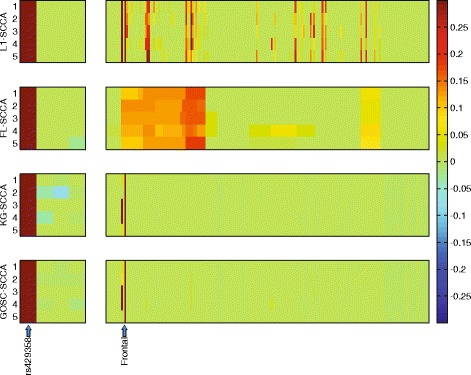
Canonical loadings estimated on the real dataset. Each row corresponds to a SCCA method: (1) L1-SCCA. (2) FL-SCCA. (3) KG-SCCA. (4) GOSC-SCCA. For each row, the estimated weights of **u** are shown on the left figure, and those of **v** on the right

Figure 2 contains the estimated canonical loadings obtained from 5-fold cross-validation. To facilitate the interpretation, we employ the heat map for this real data. Each row denotes a method, and **u** (genetic markers) is shown on the left panel and **v** (imaging markers) is on the right. As we can see, on the genetic side, all four SCCA exhibit similar canonical loading pattern. Since every SCCA here incorporates the lasso (*ℓ*_1_-norm), they select only the *APOE e4* SNP (rs429358), which is a widely known AD risk marker, with those irrelevant ones discarded to assure sparsity. On the imaging side, L1-SCCA identifies many signals which is hard to interpret. FL-SCCA fuses those adjacent features together due to its pairwise smoothness, which can be easily observed from the figure. But it is difficult to interpret either. GOSC-SCCA and KG-SCCA perform similarly again in this run. They both identify the imaging signals in accordance with the findings in [20]. It is easily to observe that they estimated a very clean signal pattern, and thus is easy to conduct further investigation. Recall the results in Table 3, the association between the marker rs429358 and the amyloid accumulation in the brain is relatively strong, and thus the signal can be well captured by both KG-SCCA and GOSC-SCCA. In addition, the correlations among the imaging variables and those among genetic variables are high enough so that the signs of these correlations can hardly be impeded by the noises. That is, the signs of sample correlations tend to be correctly estimated. Therefore, KG-SCCA does not suffer sign directionality issue, and so performs similarly to GOSC-SCCA. However, if some sample correlations are not very strong and their signs are mis-estimated, KG-SCCA may not work very well (see the results of the second synthetic dataset). In summary, this reveals that our method has better generalization ability, and could identify biologically meaningful imaging genetic associations.

## Discussion

In this paper, we have proposed a structured SCCA method GOSC-SCCA, which intended to reduce the estimation bias caused by the incorrect sign of sample correlation. GOSC-SCCA employed the GOSCAR (Graph OSCAR) regularizer which is an extension of the popular penalty OSCAR. The GOSC-SCCA could pull those highly correlated features together no matter that they were positively correlated or negatively correlated. We also provide a theoretical quantitative description of the grouping effect of our SCCA method. An effective algorithm was also proposed to solve the GOSC-SCCA problem and the algorithm was guaranteed to converge.

We evaluated GOSC-SCCA and three other popular SCCA methods on both synthetic datasets and a real imaging genetics dataset. The synthetic datasets consisted of different ground truth, i.e. different correlation coefficients and canonical loadings. GOSC-SCCA was capable of consistently identifying strong correlation coefficients on both training set and testing set, and either outperformed or performed similarly to the competing methods. Besides, GOSC-SCCA successfully and accurately recognized the signals which were the closest to the ground truth when compared with the competing methods.

The results on the real data showed that both GOSC-SCCA and KG-SCCA could find an important association between the *APOE* SNPs and the amyloid burden measure in the frontal region of the brain. KG-SCCA performs similarly to GOSC-SCCA on this real data largely because of the strong correlations between the variables within the genetic data, as well as those within the imaging data. In this case, the signs of the correlation coefficients between these variables tend to be correctly calculated, and so KG-SCCA does not have the sign directionality issue. On the other hand, if the correlations among some variables are not very strong, the performance of KG-SCCA can be affected by the mis-estimation of some correlation signs. In this case, GOSC-SCCA, which is designed to overcome the sign directionality issue, is expected to perform better than KG-SCCA. This fact has already been validated by the results of the second synthetic dataset.

The satisfactory performance of GOSC-SCCA, coupled with its theoretical convergence and grouping effect, demonstrates the promise of our method as an effective structured SCCA method in identifying meaningful bi-multivariate imaging genetic associations. The following are a few possible future directions. (1) Note that the identified pattern between the *APOE* genotype and amyloid deposition is a well-known and relatively strong imaging genetic association. Thus one direction is to apply GOSC-SCCA to more complex imaging genetic data for revealing novel but less obvious associations. (2) The data tested in this study is brain wide but targeted only at *APOE* SNPs. Another direction is to apply GOSC-SCCA to imaging genetic data with higher dimensionality, where more effective and efficient strategies for parameter tuning and cross-validation warrant further investigation. (3) The third direction is to employ GOSC-SCCA as a knowledge-driven approach, where pathways, networks or other relevant biological knowledge can be incorporated in the model to aid association discovery. In this case, comparative study can also been done between GOSC-SCCA and other state-of-the-arts knowledge-guided SCCA methods in bi-multivariate imaging genetics analyses.

## Conclusions

We have presented a new structured sparse canonical analysis (SCCA) model for analyzing brain imaging genetics data and identifying interesting imaging genetic associations. This SCCA model employs a regularization item based on the graph octagonal selection and clustering algorithm for regression (GOSCAR). The goal is twofold: (1) encourage highly correlated features to have similar canonical weights, and (2) reduce the estimation bias via removing the requirement of pre-defining the sign of the sample correlation. As a result, it could pull highly correlated features together no matter whether they are positively or negatively correlated. Empirical results on both synthetic and real data have demonstrated the promise of the proposed method.
